# Cytosolic Acidification Is the First Transduction Signal of Lactoferrin-Induced Regulated Cell Death Pathway

**DOI:** 10.3390/ijms20235838

**Published:** 2019-11-20

**Authors:** María T. Andrés, Maikel Acosta-Zaldívar, Jessica González-Seisdedos, José F. Fierro

**Affiliations:** 1 Laboratory of Oral Microbiology, University Clinic of Dentistry (CLUO), and Department of Functional Biology (Microbiology), Faculty of Medicine, University of Oviedo, 33006 Oviedo, Asturias, Spain; maikel.acostazaldivar@childrens.harvard.edu (M.A.-Z.); jessiglezsama@gmail.com (J.G.-S.); 2 Present Address: Division of Infectious Diseases, Boston Children’s Hospital/Harvard Medical School, Boston, MA 02115, USA

**Keywords:** lactoferrin, *Candida albicans*, potassium efflux, cytosolic acidification, regulated cell death, apoptosis-like, cell signaling pathway

## Abstract

In yeast, we reported the critical role of K^+^-efflux for the progress of the regulated cell death (RCD) induced by human lactoferrin (hLf), an antimicrobial protein of the innate immune system that blocks Pma1p H^+^-ATPase. In the present study, the K^+^ channel Tok1p was identified as the K^+^ channel-mediating K^+^-efflux, as indicated by the protective effect of extracellular K^+^ (≥30 mM), K^+^-channel blockers, and the greater hLf-resistance of *TOK1*-disrupted strains. K^+^-depletion was necessary but not sufficient to induce RCD as inferred from the effects of valinomycin, NH_4_Cl or nigericin which released a percentage of K^+^ similar to that released by lactoferrin without affecting cell viability. Cytosolic pH of hLf-treated cells decreased transiently (~0.3 pH units) and its inhibition prevented the RCD process, indicating that cytosolic acidification was a necessary and sufficient triggering signal. The blocking effect of lactoferrin on Pma1p H^+^-ATPase caused a transitory decrease of cytosolic pH, and the subsequent membrane depolarization activated the voltage-gated K^+^ channel, Tok1p, allowing an electrogenic K^+^-efflux. These ionic events, cytosolic accumulation of H^+^ followed by K^+^-efflux, constituted the initiating signals of this mitochondria-mediated cell death. These findings suggest, for the first time, the existence of an ionic signaling pathway in RCD.

## 1. Introduction

Human lactoferrin (hLf) is an antimicrobial protein of the innate immune system that induces a regulated cell death (RCD) in *Candida albicans*, a commensal yeast that is also an important human opportunistic pathogen [1,2,3]. In yeast, hLf-induced RCD shares many of the apoptotic cell death characteristics observed in metazoan cells, and for this reason was previously identified as an apoptosis-like process [2,4]. We previously demonstrated that potassium efflux (K^+^-efflux) is a critical step for the progress of RCD induced by lactoferrin, as it is in higher eukaryotic cells [2]. Furthermore, we identified Pma1p H^+^-ATPase (type P_3A_) as the molecular target of lactoferrin on the plasma membrane, the first reported RCD receptor in fungi [4]. Because the cytosolic accumulation of K^+^ depends on the electrochemical gradient generated by the plasma membrane Pma1p H^+^-ATPase, we hypothesized that the blocking effect of lactoferrin on this ATPase caused a sequential perturbation of ionic homeostasis [5,6,7]. This ionic perturbation, which mainly included H^+^ and K^+^ ions, occurred at the cytoplasmic and mitochondrial level leading to regulated cell death. In this process, we reported the central role of mitochondria, specifically ATP synthase, for the subsequent progress of RCD induced by lactoferrin [4,8].

Potassium efflux, mediated by a wide variety of K^+^ channels, has been well documented in many apoptotic model systems using multicellular and unicellular eukaryotes but the underlying mechanism(s) remain largely elusive [9,10,11,12,13,14,15,16,17,18,19,20,21,22]. Moreover, it is unknown why K^+^-depletion is critical for progress on the apoptotic pathway. Low intracellular potassium could favor cell death by initiating various pro-apoptotic processes such as activation of intracellular enzymes, and hyperpolarization of the plasma membrane and osmotic lysis of vacuoles [12,17,23]. These proposals imply that K^+^-efflux is a sufficient condition to trigger the RCD process, an idea that is apparently supported by different observations made both in higher eukaryotic cells and in yeasts, in which blocking of the outward K^+^ channels prevents regulated cell death [2,14,24,25].

In parallel, cytosolic acidification is also an early event observed in apoptosis of eukaryotic cells, including yeasts [26,27,28]. Rapid and transient changes of cytosolic pH may act as intracellular signals of important cellular functions [29,30,31]. However, the role of cytosolic acidification in regulated cell death has not been explored. It is thought that the ionic imbalance during the RCD process is a consequence of the structural and functional alterations experienced by the dying cell rather than early signals of a putative intracellular signaling pathway [14].

The above arguments raise the question of whether K^+^- and H^+^-flux represent intermediate signals required for the induction of RCD and whether they are causally linked. The aim of this study was to identify the intracellular signal responsible for RCD activation in response to the blocking effect of human lactoferrin on Pma1p H^+^-ATPase. For that purpose, we used a simplified experimental system, which included: (*i*) a unicellular eukaryote (*C. albicans*), (*ii*) a target-specific inducer of regulated cell death process (lactoferrin), and *iii*) a salt-free buffer. Using these minimal elements and restricted test conditions, current work focused on elucidating the first ionic cytosolic events related to RCD and was undertaken with the following objectives: (a) to evaluate whether inhibition of lactoferrin activity by extracellular K^+^ would result from either cation- and/or osmotic-induced changes in the microorganism or by inhibition of the protein–target interaction; (b) to identify the K^+^-channel putatively involved in the RCD process induced by lactoferrin; (c) to determine whether cellular K^+^-efflux is a sufficient signal to initiate RCD or merely a consequence of other previous events; and in this latter case, (d) to identify possible upstream event/s of the RCD pathway promoting flow of K^+^ out of the cell (K^+^-efflux).

The understanding of the early signaling function of cytosolic ions in the RCD sequence and its possible extrapolation to higher eukaryotic cells may lead to therapeutic applications in the future and new concepts.

## 2. Results

### 2.1. Effect of Extracellular Factors on the RCD Induced by Lactoferrin

**Figure 1:**
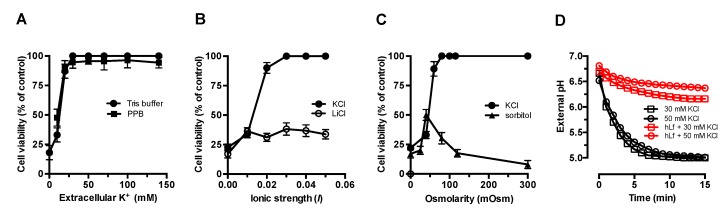
Influence of extracellular factors on lactoferrin-induced regulated cell death. *C. albicans* cells (10^5^ cells/mL) were incubated with 5 μM lactoferrin (hLf) for 90 min at 37 °C in the presence of (**A**) different extracellular K^+^ concentrations in Tris buffer (circles) or potassium phosphate buffer (PPB, squares); (**B**) different ionic strength calculated in the presence of KCl (solid circles) and LiCl (open circles) solutions; or (**C**) equivalent osmolarity values of KCl (circles) or sorbitol (triangles) solutions. The cell viability was determined by a plate-count method. (**D**) Effect of extracellular K^+^ on the interaction of lactoferrin with the H^+^-ATPase. The H^+^-extrusion mediated by the plasma membrane Pma1p H^+^-ATPase of *C. albicans* cells suspended in 30 mM KCl (squares) or 50 mM KCl (circles) was determined in the presence (red lines) or in the absence (black lines, control) of lactoferrin by monitoring glucose-induced acidification of the external medium. The results are the means ± SD from duplicates of at least three independent experiments. In Figure 1D, only the mean data (*n* = 3) are shown, and the bars representing standard errors (coefficient of variation of <10%) are omitted for clarity.

In previous reports, we showed that the candidacidal effect caused by lactoferrin is a type of regulated cell death (i.e., apoptosis-like process) that can be prevented by the presence of extracellular K^+^ [2,32]. Lactoferrin-treated cells suspended in Tris buffer or potassium phosphate buffer (10 mM K_2_HPO_4_-KH_2_PO_4_, pH 7.4), containing different concentrations of KCl, were fully protected from the lactoferrin killing activity at ≥30 mM potassium (Figure 1A). The comparison of the inhibition patterns obtained in each of these different buffers showed a similar dependence of the K^+^ concentration and indicated that only extracellular K^+^ was involved in the inhibition of the candidacidal activity of lactoferrin.

It is known that the properties of the microbial cell wall and other experimental factors can influence the susceptibility to many cationic antimicrobial peptides [33]. Therefore, we evaluated whether cellular protection mediated by extracellular K^+^ was a consequence of either the ionic strength or the osmotic conditions used. Figure 1B shows the data of the killing assays performed in KCl or LiCl solutions adjusted to different ionic strengths (*I*). In the presence of K^+^, the candidacidal activity of lactoferrin was inhibited by 93% ± 5% and 100% by *I* values of 0.02 and 0.03, respectively. In the presence of Li^+^, used to obtain *I* values similar to those of KCl solutions, only partial protection (≤36% cell survival) was observed in hLf-treated cells to *I* ≥ 0.01, suggesting an unknown alternative mechanism. However, the cell viability patterns obtained in the presence of these monovalent cations were clearly different, showing that the candidacidal activity of lactoferrin was abrogated by *I* ≥ 0.03 in the presence of K^+^.

To determine whether the protective effect of extracellular K^+^ was due to extracellular osmotic conditions, we tested lactoferrin activity under equivalent osmolarity values KCl and sorbitol, a non-ionic osmotic stabilizer. Although partial inhibition was observed at lower osmolarity values of sorbitol (≤80 mOsm), higher osmolarity values, which were equivalent to ≥20 mM KCl, were not inhibitory with respect to the control assays performed in Tris buffer alone. For example, 300 mOsm sorbitol, comparable in osmotic strength with 150 mM KCl or 140 mM NaCl, was unable to inhibit the regulated cell death induced by lactoferrin (Figure 1C). Therefore, the activity of lactoferrin was not significantly modified by different osmotic conditions associated with extracellular K^+^ concentration over a wide range of concentrations.

Lactoferrin blocks Pma1p H^+^-ATPase by preventing the extrusion of cytosolic protons out of the cell membrane, an effect that can easily be observed by a glucose-induced proton extrusion test [4]. In glucose-induced extracellular acidification assays, the blocking effect of hLf on Pma1p H^+^-ATPase remained unchanged in the presence of K^+^ concentrations (30 and 50 mM) that inhibit the candidacidal activity of lactoferrin (Figure 1D).

These results suggest that the inhibitory effect of extracellular K^+^ (≥30 mM) on lactoferrin-induced regulated cell death was not due to the experimental conditions used, such as low ionic strength and osmolarity, nor to other factors that could prevent the interaction between Pma1p H^+^-ATPase and lactoferrin.

### 2.2. Identification of K^+^ Transporters in the RCD Process

**Figure 2:**
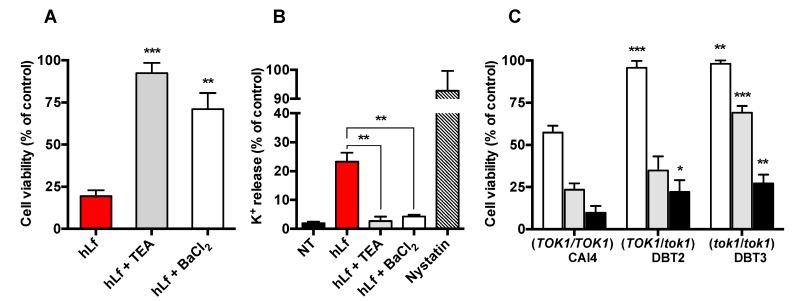
Identification of K^+^-channel involved in the lactoferrin-induced RCD process. (**A**) Viability of yeast cells (10^5^ cells/mL) suspended in Tris buffer and treated with 5 μM lactoferrin (hLf, red column) or pre-incubated for 15 min at 37 °C with the K^+^-channel inhibitors tetraethylammonium (10 mM TEA; gray column) or Ba^2+^ (10 µM BaCl_2_; white column) before the addition of 5 μM hLf. (**B**) Potassium efflux measured from non-treated cells (NT) assayed under the above conditions (black column; negative control), treated with hLf alone (red column) or pre-incubated with 10 mM TEA (gray column) or 10 µM BaCl_2_ (white column) before the addition of lactoferrin. Nystatin (100 μg/mL) was used as a positive control of K^+^ released by permeabilized cells (dashed column). (**C**) Antifungal activity of three concentrations of hLf on *C. albicans TOK1*-disrupted strains. The strains (10^5^ cells/mL) were incubated for 90 min at 37 °C with 1.25 μM (white column), 2.5 μM (gray column), or 5 μM (black column) of lactoferrin. All the percentages of viability and K^+^ released correspond to cells suspended in Tris buffer and incubated with hLf for 90 min at 37 °C. The cell viability was determined by a plate-count method. The results are the means ± SD from duplicates of at least three independent experiments. Statistical significance was assessed by Student’s *t* test. * *p* < 0.05; ** *p* < 0.01; *** *p* < 0.001.

The efflux of potassium is a critical event for the RCD progress induced by lactoferrin, and we have previously showed that tetraethylammonium (TEA) and 4-aminopyridine (4-AP), blockers of voltage-gated K^+^-channels, prevented the candidacidal activity of this cell membrane depolarizing protein [2,34]. Figure 2A shows that *C. albicans* cells pre-incubated in Tris buffer containing 10 mM TEA exhibited a high lactoferrin resistance compared to cells treated only with lactoferrin (92% ± 6% versus 19% ± 4% cell survival, respectively). Similarly, cells pre-incubated in the presence of Ba^2+^ (10 µM BaCl_2_), a selective inhibitor of the outward K^+^-channel Tok1p of *C. albicans*, were significantly more resistant to lactoferrin (71% ± 8% versus 19% ± 4% cell survival, respectively) [35]. After 90 min, potassium released from lactoferrin-treated cells pre-incubated with either TEA or BaCl_2_ was substantially lower than what K^+^ released from cells exposed only to lactoferrin or nystatin (Figure 2B). The latter is a partially selective pore-forming agent in the plasma membrane of fungi that mainly releases ions and small molecules from cytosol.

The preventive effect of TEA and Ba^2+^ supported the participation of Tok1p channels for the progress of the cell death pathway induced by lactoferrin. To assess this possibility, the susceptibility to lactoferrin of *TOK1*-disrupted strains *C. albicans* DBT2 (*TOK1*/*tok1*) and DBT3 (*tok1*/*tok1*) was compared with that of their parental strain *C. albicans* CAI4 (*TOK1*/*TOK1*), the strain expressing both wild-type alleles of *TOK1* functional. Figure 2C shows that the strains DBT2 and DBT3 were significantly more resistant than the strain CAI4 (95% ± 4% and 98% ± 2% versus 57% ± 8% cell survival, respectively) to 1.25 µM lactoferrin. The involvement of the Tok1p channels in the cell death was also reflected in the different susceptibility to lactoferrin of both modified strains (Figure 2C). The strain DBT3, without functional *TOK1* alleles, was substantially more resistant with respect to the DBT2 strain with one functional *TOK1* allele (35% ± 9% versus 69% ± 7% cell survival, respectively). Thus, two *TOK1* disruptions doubled the viability increment produced by a single disruption when these *TOK1*-disrupted strains were exposed to 2.5 µM lactoferrin (Figure 2C).

An examination of all the above data indicate that, under our experimental conditions, the K^+^-efflux in the presence of lactoferrin, a protein that causes plasma membrane depolarization, was mediated by the Tok1p channel, a voltage-gated K^+^ channel that is activated only by plasma membrane depolarization [32,36,37].

### 2.3. Effect of Cellular K^+^ Depletion on RCD Triggering

Lactoferrin induces a RCD in yeast which is accompanied of K^+^-efflux during the first 20 min of treatment [34]. To determine if cellular K^+^ depletion is a sufficient signal for activation of the regulated cell death pathway, *C. albicans* cells were exposed to different chemical agents that decrease the intracellular K^+^ concentration by different mechanisms of action.

#### 2.3.1. Effect of Valinomycin-Mediated K^+^ Release on Cell Viability

**Figure 3:**
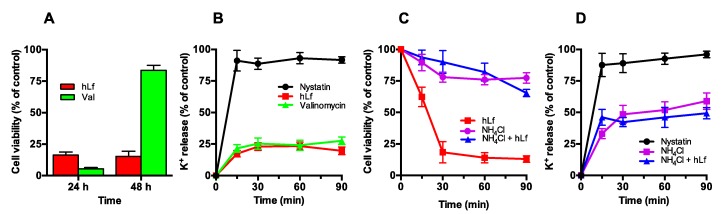
Effect of different K^+^-depletion inducers on cell viability. (**A**) Antifungal activity of lactoferrin and valinomycin. The cells (10^5^ cells/mL) were incubated in Tris buffer for 90 min at 37 °C with 5 µM lactoferrin (hLf, red column) or 50 µg/mL valinomycin (Val; green column) and cell viability was calculated at 24 h and 48 h. (**B**) Time-course of K^+^ release induced by lactoferrin and valinomycin. The K^+^ released was measured at different times after the addition of 20 µM lactoferrin (red line), 50 µg/mL valinomycin (green line), or 100 µg/mL nystatin (black line, positive control) to cell suspensions (10^7^ cells/mL). (**C**) Effect of NH_4_Cl on viability of lactoferrin-treated cells. The time-kill curve of cells (10^5^ cells/mL), pre-incubated in the presence of 10 mM NH_4_Cl and treated with (blue line) or without 5 µM hLf (purple line) to determine cell viability. Lactoferrin-treated cells in the absence of NH_4_Cl (red line) were used as control. (**D**) Time-course of K^+^-release in the presence of NH_4_Cl. Cell suspensions were treated with (blue line) or without 20 µM hLf (purple line) in the presence of 10 mM NH_4_Cl. The K^+^ release was determined at different times of the incubation period performed at 37 °C. Nystatin (100 µg/mL) was used as positive control in the measurements of released K^+^. Each value shown is the mean ± SD from duplicates of at least three independent experiments.

Cells were incubated (90 min at 37 °C) with valinomycin (50 µg/mL), a K^+^-selective ionophore, or 5 μM lactoferrin (positive control), and cell viability was determined after 24, 48 and 72 h by a plate-count method. After incubating the plates for 24 h, the viability of the cells treated with valinomycin or lactoferrin significantly decreased (5% ± 2% and 15% ± 4% cell survival, respectively), a result compatible with a candidacidal effect for both agents (Figure 3A). However, the same plates containing the valinomycin-treated cells showed late growth, forming small colonies (83% ± 7% cell survival) after a 48-h incubation. These colonies, delayed in their growth with respect to the control (cells not treated with valinomycin), reached a complete development at 72 h. Conversely, no recovery of viability of lactoferrin-treated cells was observed even after 72 h of incubation (16% ± 2% cell survival), a congruent result for a protein that induces a RCD process.

We interpreted that cells incubated with valinomycin alone, suspended in K^+^-free media (Tris buffer), were depleted of K^+^ ions to an intracellular level non compatible with cell viability. These apparently non-viable but vital cells restored their intracellular K^+^ concentration after they were spread onto SDA plates, where K^+^ uptake was restored and the concentration of valinomycin present in the inoculum was diluted by diffusion in the solid medium, thus recovering their cell viability but showing a delay in the formation of colonies with respect to the control.

To investigate whether the differences of antifungal activity of valinomycin and lactoferrin could be explained by possible differences in their ability to deplete intracellular K^+^ ions, the K^+^ released in the presence of each of these compounds was compared with respect to the total cellular K^+^ content (100%). Interestingly, valinomycin- and lactoferrin-treated cells released a similar percentage of K^+^ (25% ± 4% and 23% ± 3%, respectively) with a similar kinetic release pattern (Figure 3B). A progressive potassium release was detected during the first 20 min, further remaining without changes. The similarity in the percentage and releasing-K^+^ pattern of valinomycin- and lactoferrin-treated cells, compared with the total cellular K^+^ content and the percentage of K^+^ released by nystatin-treated cells, suggested that K^+^-efflux was electrogenic.

#### 2.3.2. Effect of NH_4_Cl-Mediated K^+^ Extrusion on Cell Viability

It was previously reported that yeast cells at pH 8.0 only extrude cellular K^+^ (>50%) in the presence of ammonium chloride [38]. The loss of intracellular K^+^ was described as the consequence of the rapid cytosolic alkalinization induced by extracellular NH_4_^+^ [38,39]. This cationic molecule is then converted to electroneutral NH_3_ at alkaline pH and diffuses into cells where it binds to free cytosolic protons. Subsequently, the cytosolic alkalinization is compensated by a homeostatic response involving the exchange of intracellular K^+^ by H^+^, to restore the initial cytosolic pH values [38].

In our assays, the viability of cells treated with 10 mM NH_4_Cl was not substantially modified with respect to untreated cells (Figure 3C). However, the release of K^+^ from NH_4_Cl-treated cells was approximately 54% of total intracellular K^+^ after 90 min (Figure 3D). Then, the release of K^+^ in NH_4_Cl-treated cells was twice that observed in lactoferrin or valinomycin-treated cells without this greater loss of K^+^ substantially affecting their viability.

#### 2.3.3. Effect of Nigericin-Mediated K^+^/H^+^ Exchange on Cell Viability

The lipophilic ionophore nigericin mediates electroneutral antiport of K^+^ and H^+^ across biological membranes, thus mimicking the intracellular ionic context caused by lactoferrin. Nigericin-induced regulated cell death of *S. cerevisiae* cells with a declining intracellular pH was previously reported [40]. In a similar way, the viability of nigericin-treated *C. albicans* cells was estimated as 53%, a significant loss of viability with respect to untreated cells.

#### 2.3.4. Effect of Piericidin A on the Lactoferrin Induced K^+^-efflux

The lactoferrin-treated cells were pre-incubated with 16 µM piericidin A for 15 min at 37 °C to inhibit the complex I of the respiratory chain of *C. albicans* cells. Measurement of the K^+^ released from cells pre-incubated with piericidin A showed a similar percentage of K^+^ released with respect to that measured from cells treated with lactoferrin alone (27% versus 24% of K^+^ released, respectively). However, piericidin A is an effective protector of lactoferrin-induced RCD, as we previously reported [4], indicating again that K^+^-efflux alone is not sufficient to initiate RCD and that this critical step could be a consequence of a previous signaling event related to proton balance.

### 2.4. Evaluation of the Role of Cytosolic pH in Regulated Cell Death Triggering

Since lactoferrin inhibits Pma1p H^+^-ATPase, an early accumulation of protons in the cytosol of metabolically active cells is expected under our experimental conditions. This raises the question of whether changes in intracellular pH could represent, in our case, the first intracellular signal of a hypothetical signaling pathway of cell death.

**Figure 4:**
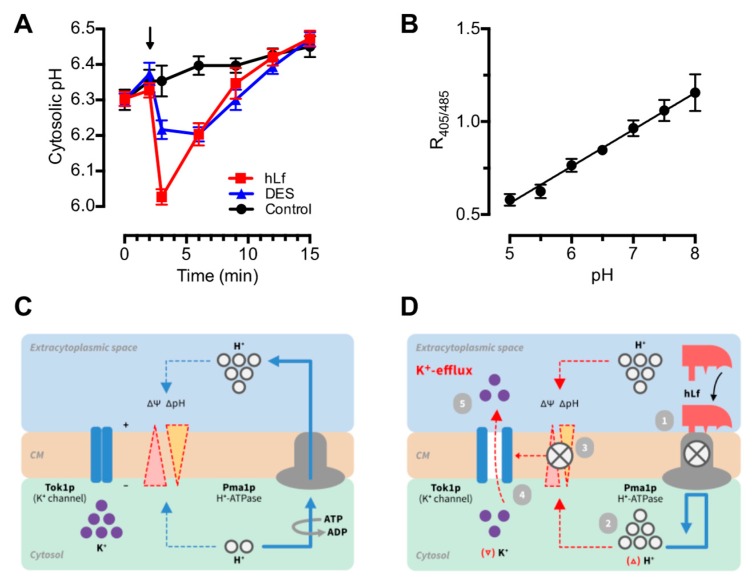
Effect of lactoferrin on cytosolic pH. (**A**) Transient cytosolic acidification induced by lactoferrin. Cytosolic pH of *C. albicans*-adapted pHluorin cells (black line, control), exposed to 5 µM lactoferrin (red line) or 25 µM diethylstilbestrol (DES, blue line), was determined at different times. These inhibitors of fungal Pma1p H^+^-ATPase were added to the cell suspensions at the indicated time (arrow). Cytosolic pH values were derived from fluorescence intensity measurements at different times over 15 min and calculated according to the calibration curve. (**B**) Calibration curve showing measured ratio of fluorescence intensity at 405 nm to intensity at 485 nm versus pH of pHluorin-expressing permeabilized cells equilibrated in buffers of increasing pH. Data are shown as means ± SD from duplicates of at least three independent experiments. (**C**) The normal activity of Pma1p H^+^-ATPase of cytoplasmic membrane (CM) generates a proton gradient (ΔpH) and an electrical (Δ*Ψ*) gradient (negative inside) indicated by dashed lines. Cytosolic K^+^ is retained by the negative electrical charge. (**D**) The figure shows the sequence of linked events induced by lactoferrin (i.e., cytosolic acidification and K^+^-efflux). The blocking effect of lactoferrin on Pma1p H^+^-ATPase (1) causes a transient accumulation of protons in cytosol, detected as cytosolic acidic pH shift (2), and the subsequent loss of electrochemical gradient (3). As a consequence, the voltage-dependent channel Tok1p opens upon plasma membrane depolarization (4) allowing K^+^-efflux (5).

Monitoring intracellular pH by applying the ratiometric pHluorin method in the *C. albicans* JKC1562 strain showed a rapid drop in cytosolic pH estimated in approximately 0.3 pH units (6.33 to 6.02) after approximately 1 min of lactoferrin addition. This acidic pH shift was followed by a slow but progressive cytosolic alkalinization that reached the initial pH value (pH 6.33) approximately 6 min later (Figure 4). In a similar way, diethylstilbestrol (DES), a known Pma1p H^+^-ATPase specific blocking agent, elicited a rapid but weaker response compared with the transient cytosolic acidification induced by lactoferrin (Figure 4).

## 3. Discussion

Accumulated evidence indicates that reduced concentrations of intracellular K^+^ provide a permissive cytosolic environment for apoptosis and that elevation of extracellular K^+^ prevents this type of regulated cellular death (RCD) in higher eukaryotic cells and yeast [2,12,14]. Similarly, K^+^-efflux is a critical stage for the progress of the RCD process induced in yeast by lactoferrin and was also observed with different inducers of RCD in fungi such as acetic acid, chlorogenic acid, glucose, *Penicillium chrysogenum* antifungal protein, and sorbic acid indicating also its importance in the RCD process of lower eukaryotic cells [17,21,41,42,43]. However, an explanation of the intracellular mechanism(s) by which cellular K^+^-efflux occurs and why this event is critical for the progress of the RCD cascade remains to be elucidated [13,14,15].

Previous work demonstrated that lactoferrin targets Pma1p H^+^-ATPase, inducing a regulated cell death, mediated by K^+^-efflux, that was inhibited by extracellular K^+^ by unknown mechanism/s [2,32]. Since lactoferrin is a cationic glycoprotein (pI ~8.7) at assayed pH (pH 7.4), the loss of activity could be due to a competitive effect of K^+^ cations for the lactoferrin-binding sites shielding them from this antimicrobial factor. However, our data from time-kill experiments show that the preventive effect of extracellular K^+^ (≥30 mM) was not related to decreased cell binding of lactoferrin nor other cellular changes associated to the experimental conditions. Therefore, we reasoned that the flow of K^+^ was an ionic homeostatic response to the blocking effect of lactoferrin on Pma1p H^+^-ATPase. In this respect, we assumed that the reported plasma membrane depolarization of hLf-treated cells was partially due to the dissipation of the proton gradient (ΔpH) caused by the cytosolic accumulation of H^+^, after Pma1p H^+^-ATPase inhibition. In turn, the electrical negative force that retained intracellular K^+^ was eliminated and positively charged K^+^ ions passively flowed out of the cell through specific outward K^+^ channels, yielding a measurable K^+^-efflux [4,32]. To further evaluate this idea, we first performed the identification of the putative K^+^-channels related with this early apoptotic event.

In yeast, the cation transporters Nha1, Ena1-4, and Tok1p are the only known channels involved in the K^+^-efflux [7,44]. The activity of Nha1, a Na^+^ (K^+^)/H^+^ antiporter, is dependent on the proton gradient generated by the Pma1p H^+^-ATPase, blocked by lactoferrin in our assays. Moreover, the ability of Ena1-4, a Na^+^ (K^+^) transport ATPase, to extrude K^+^ has not been reported for *Candida* genus. Consequently, the involvement of Nha1 and Ena 1-4 in the K^+^-efflux induced by lactoferrin was excluded. The only outward-rectifying K^+^-channel present in the plasma membrane of yeast cells is Tok1p, a voltage-dependent outward rectifier channel that releases K^+^ exclusively when activated by membrane depolarization [36,45]. The Tok1p channel is blocked by TEA^+^ and Ba^2+^ cations [45]. *C. albicans* cells pre-incubated with TEA or BaCl_2_ were significantly more resistant to lactoferrin compared to the non-treated cells, and this was correlated with a clear decrement of K^+^ efflux. In a similar way, *C. albicans TOK1*-disrupted strains (*TOK1*/t*ok1*, and *tok1*/*tok1*) exhibit an increased resistance to low concentrations of lactoferrin. These results indicated that, under our experimental conditions, the K^+^-efflux was mediated by the Tok1p channel. To our knowledge, this is the first report identifying a K^+^-channel involved in the regulated cell death of fungi.

It is noteworthy that the analysis of the data of the K^+^-efflux of cells treated with lactoferrin showed a kinetic pattern, similar to that obtained using valinomycin alone, compatible with an electrogenic flow through the plasma membrane. It seems that K^+^ ions left the cell through Tok1p channels driven by its concentration gradient but generating behind an unbalanced electric charge (negative inside), which prevented a greater K^+^-efflux. This could explain two of our previous results obtained using hLf-treated cells [4,34]: (a) the relatively small percentage of K^+^ released from the cells (approximately 25% of total K^+^ cellular content), and (b) the protective effect of external K^+^ on the candidacidal activity of lactoferrin. In the latter case, the complete dissipation of K^+^-gradient occurred when extracellular K^+^ was ≥30 mM, avoiding the K^+^-efflux and the subsequent cell death.

The above results suggest that preservation of intracellular K^+^, due to Tok1p blocking or to the loss of K^+^ gradient, prevented the regulated cell death. In this case, the question arises whether only K^+^-efflux could be sufficient to activate RCD.

If the loss of intracellular K^+^ is a necessary and sufficient signal for a cell to undergo regulated cell death, then chemical induction of K^+^ depletion should trigger a similar RCD process. To test this suggestion, *C. albicans* cells were exposed to three chemical agents (i.e., valinomycin, NH_4_Cl, nigericin) which promote a clear decrease in intracellular K^+^ concentration by different mechanisms. The neutral ionophore valinomycin forms plasma membrane permeant complexes with intracellular K^+^ (valinomycin-K^+^) which exhibit a positive charge. Consequently, an outward movement of valinomycin-K^+^ complexes across the plasma membrane is generated. In our experimental conditions, the transmembrane flux of valinomycin-K^+^ complexes was only dependent on the K^+^ gradient, but limited by the electrical gradient (negative inside) generated by the outward flux of valinomycin-K^+^. Supporting this scenario, the determination of extracellular K^+^ from valinomycin- and hLf-treated cell suspensions showed similarity in both efflux kinetics and percentage of K^+^ released (approximately 25%), suggesting that K^+^-efflux induced by lactoferrin was electrogenic. Surprisingly, valinomycin-treated cells were unable to undergo a regulated cell death, and only a delayed growth of the colonies was observed. Although the valinomycin effect on other cell organelles was not evaluated, the above data are consistent with our previously reported observations and hypothesis, suggesting that the observed K^+^-efflux in hLf-treated cells is a necessary step for the progress of the RCD pathway but it is not the essential activator of the RCD process [4,32].

According to these results and in order to clarify the role of K^+^ depletion in the activation of the RCD sequence, the cells were also exposed to ammonium chloride (NH_4_Cl), a chemical compound that promotes a high K^+^-efflux concomitantly with a cytosolic alkalization [38]. In the presence of NH_4_Cl, *C. albicans* cells suspended in alkaline medium (pH 8.0) underwent a high K^+^ release, higher than that induced by lactoferrin alone and in a similar percentage to that previously reported for *S. cerevisiae* cells exposed to NH_4_Cl [38]. Despite the high percentage of K^+^ released (approximately 54%), the viability of the NH_4_Cl-treated cells remained unchanged, supporting again that the depletion of K^+^ alone was not a sufficient intracellular signal to initiate the RCD process. Moreover, this result suggested that the protective effect of NH_4_Cl observed on hLf-treated cells could be associated with its alkalizing effect on the cytosol rather than with its ability to induce a high K^+^ release. Furthermore, the suggested importance of cytosolic pH as an initiator of the cell death process was supported by the important loss of cell viability (approximately 53% of cell survival) caused by the K^+^/H^+^ ionophore nigericin that facilitates K^+^ depletion by allowing an H^+^ influx, leading to acidification of cytosol.

Previously, we reported that RCD induced by lactoferrin is blocked by piericidin A, an inhibitor of the respiratory complex I of *C. albicans* cells [4]. Here, our data indicated that K^+^-efflux induced by lactoferrin was not modified in cells pre-incubated with piericidin A, suggesting that: (a) The K^+^-efflux precedes the previously reported mitochondrial events involved in this RCD process; and (b) Pro-apoptotic intracellular events activated by K^+^-depletion, such as activation of hydrolytic enzymes, vacuolar collapse and hyperpolarization of the plasma membrane, are not involved in this type of RCD [4,9,12,17,23].

All these findings together with our previously reported observations underscore the importance of K^+^-efflux *ab initio* of the RCD induced by lactoferrin, but clearly show that this early event is not sufficient to initiate this cell death pathway [2,4,32]. Given all these assertions, we reasoned that cytosolic acidification caused by the rapid blocking effect of lactoferrin on the Pma1p H^+^-ATPase could be the triggering signal of this type of RCD [4].

Cytosolic acidification is an early apoptotic event observed in cells from multicellular and unicellular eukaryotic organisms exposed to different apoptogenic stimuli [26,27,28,31]. Therefore, we address the question of whether the decrease of cytosolic pH could trigger the RCD process induced by lactoferrin. In this regard, the kinetic measurement of intracellular pH using pHluorin provided three interesting features of this event: (a) Cytosolic acidification was the first detectable event (≤1 min after addition of the lactoferrin) associated to RCD; (b) This rapid drop of intracellular pH (acidic pH shift) was estimated in approximately 0.3 pH units at 1 min, a pH value similar to that previously reported for apoptosis of higher eukaryotic cells [26,28,31]; and (c) Remarkably, the cytosolic acidification induced by lactoferrin was transitory, recovering the initial pH value after approximately 7 min of the addition of lactoferrin. These data provided clear evidence of a rapid change in cytosolic pH as a result of an external stimulus, the specific interaction of lactoferrin with Pma1p H^+^-ATPase. It should be noted that the kinetics of the assays performed to evaluate K^+^-efflux and cytosolic acidification showed that the acidic pH shift preceded the K^+^-efflux, a finding that is consistent with our previous results arguing against the role of K^+^-efflux as a trigger for this RCD. Both cytosolic events (i.e., cytosolic acidification and K^+^-efflux), consequence of the lethal *pas-de-deux* of Pma1p H^+^-ATPase and Tok1p channels, were the earliest activation signals of the RCD sequence. Inhibition of only one of these events was sufficient to inhibit lactoferrin-induced cell death, as observed when cells were pre-incubated with NH_4_Cl or K^+^-channel-blocking agents.

There are previously reported data that apparently support the idea that cytosolic acidification is an inducing signal of cell death in fungi. For example, many of the RCD inductors in fungi are weak acids or acidic compounds, such as acetic acid, acetylsalicylic acid, formic acid, valproic acid, propionic acid, and chlorogenic acid [21,46,47,48,49,50,51]. These compounds rapidly access the cytosol to be dissociated into protons and their counterions, causing a drop of cytosolic pH that may be accompanied by K^+^-efflux, as previously reported [21,41,43]. In all these cases, it could be assumed that the continuous entry of a weak acid into the cytosol overwhelms the regulatory mechanisms of intracellular pH. If so, the sudden intracellular acidification (acidic pH shift) could be a sufficient signal to initiate RCD similar to that induced by lactoferrin in our restricted experimental conditions.

In general, our results and the preceding evidence strongly support that transient acidification of the cytosol (acid pH shift ≥0.3 pH units) constitutes *per se* an RCD-inducing signal and the first intracellular step of this ion signaling pathway. Consistently, other ionic events (i.e., K^+^-efflux) seems to be a consequence of this initial perturbation of cytosolic pH.

**Figure 5:**
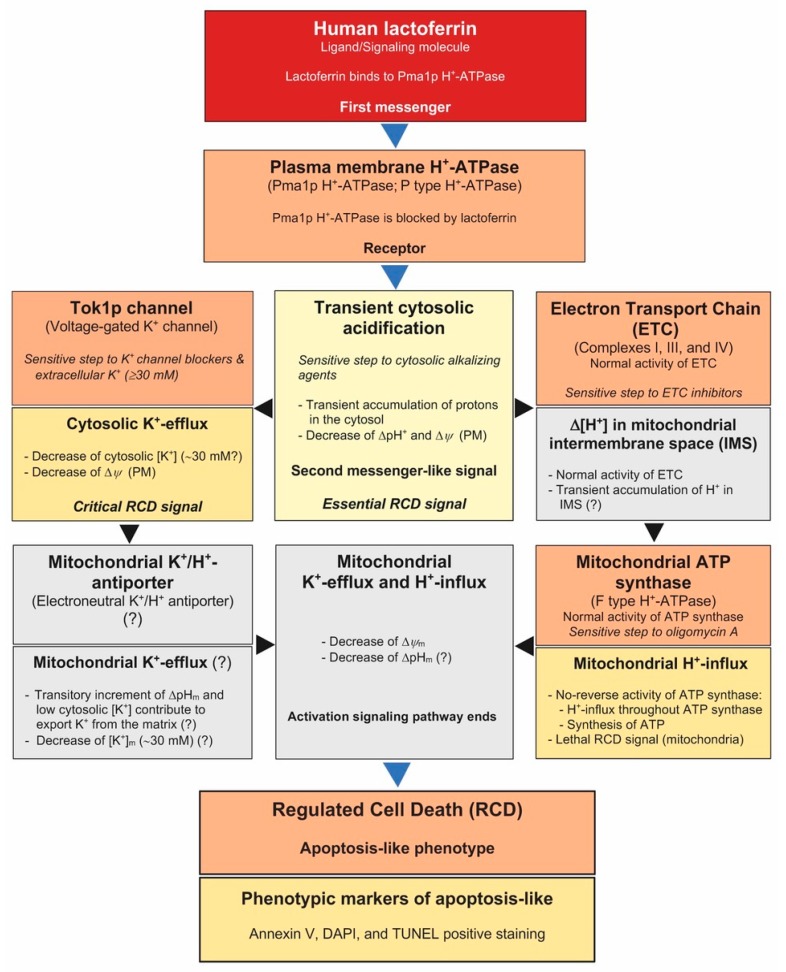
Proposed model of the RCD signaling pathway induced by lactoferrin. The scheme includes hypothetical steps (gray boxes) of this process based on our previously reported data [2,4,32,34]. In yeast cells, Pma1p H^+^-ATPase actively extrudes H^+^ out the cell and generates a proton gradient (ΔpH) and an electrical gradient (Δ*Ψ*) which allow pH regulation and the flux of other ions and nutrients across the plasma membrane (PM). The here proposed cell death signaling pathway includes a sequence of ionic events in the cytosol as response to an extracellular signal, as follows: (1) Lactoferrin (hLf, first messenger) binds to the plasma membrane Pma1p H^+^-ATPase (receptor) blocking this proton pump [4]. (2) Protons are transitorily accumulated in the cytosol, an event detected as a cytosolic acidic pH shift (second messenger-like signal), inducing a Δ*Ψ*-decrease. (3) This PM-depolarization opens the voltage-gated K^+^ channel Tok1p, allowing a passive and electrogenic K^+^-efflux [2,32,34]. (4) Hypothetically, both cytosolic K^+^ depletion and transient cytosolic acidification could favor in turn the loss of mitochondrial potassium, probably via a K^+^/H^+^ exchanger, and the simultaneous entry of H^+^ to the mitochondrial matrix, causing a loss of Δ*Ψ*_m_, as reported in [2]. The H^+^-influx to the matrix, via ATP synthase, is an essential event because inhibition of either electron transfer chain (ETC) or ATP synthase prevented the progress of this cell death pathway [2,4]. (5) The supposed perturbation of the mitochondrial homeostasis could induce further events previously visualized as phenotypic apoptotic markers [2]. Events marked with (?) are hypothetical steps that are being studied.

Cytosolic acidification was proposed as an intracellular messenger of several cell pathways associated to the metabolism and growth of eukaryotes, including yeast [29,30,31]. However, the exact role of the cytosolic accumulation of protons in RCD signaling pathways has not been studied [27,31]. In our case, it is tempting to speculate that an ion-mediated pathway is associated to lactoferrin-induced regulated cell death (Figure 5). This ionic signaling pathway is initiated by the interaction of lactoferrin (ligand, first messenger) with Pma1p H^+^-ATPase (plasma membrane receptor) of yeast to induce a transient cytosolic acidification (second messenger-like signal) that depolarizes the cell membrane. Depolarization of the membrane causes the opening of voltage-operated K^+^ channels (Tok1p), allowing K^+^-efflux. This ionic event in addition to cytosolic acidification promotes the previously reported mitochondrial response leading to RCD [4]. To the best of our knowledge, this is the first time that a transient cytosolic acidification is proposed as a triggering signal of a cell death signaling pathway.

In a previous report, we suggested that perturbation of the ‘cytosolic ionic homeostasis’ leads to an alteration of ‘mitochondrial ionic homeostasis’, and we showed that a functional H^+^-ATPase (ATP synthase), translocating protons toward mitochondrial matrix, was essential for the progress of the RCD induced by lactoferrin [4]. In our model, the sudden increase of cytosolic protons coupled to the cytosolic K^+^-efflux seems to represent concatenated signals that converge at the level of the mitochondria, favoring the simultaneous entry of protons into the mitochondrial matrix, mainly through ATP synthase, and the possible loss of mitochondrial potassium. If so, it could be suggested that the protective effect of extracellular K^+^ (≥30 mM) observed on lactoferrin-treated cells might represent a subrogated protective effect at the mitochondrial level. In such a case, the ionic equilibrium of K^+^ reached on both sides of the cytoplasmic membrane prevents the K^+^-efflux, preserving a critical cytosolic K^+^ concentration (i.e., ~30 mM). This concentration of K^+^ would be necessary to presumably prevent an equivalent loss of mitochondrial K^+^ that, otherwise, would favor the entrance of protons to the mitochondrial matrix with lethal consequences for the cell [4].

In conclusion, these findings suggest, for the first time, the existence of a signaling pathway from the cytoplasmic membrane to mitochondria where only ions act as early signaling molecules in a RCD pathway. The acidic pH shift in the cytosol activated this RCD signaling pathway, resulting in intra-mitochondrial ionic disturbance that promoted a further array of phenotypical features previously detected as apoptotic cell markers [2].

Our experimental design represents a high efficiency model for future sequential dissection of cell death pathways in fungi. Due to many of the hallmarks of the RCD process being evolutionarily well conserved events, it would be interesting to determine whether the findings reported here, obtained in an experimental system of limited complexity, may be extrapolated to the RCD processes of higher eukaryotic cells (i.e., apoptosis). If this were the case, our findings might help to design new therapeutic approaches.

## 4. Materials and Methods

### 4.1. Materials

Recombinant human apo-lactoferrin was obtained from Sigma-Aldrich (St. Louis, MO, USA) and Ventria Bioscience (Aurora, CO, USA). Diethylstilbestrol (DES), nigericin, nystatin, piericidin A, tetraethylammonium (TEA), Tris(hydroxymethyl)amino-methane (Tris), and valinomycin were purchased from Sigma-Aldrich. All chemicals used were of analytical grade and were supplied by Sigma-Aldrich. Sabouraud-2% dextrose broth (SDB) was purchased from Merck KGaA (Darmstadt, Germany).

### 4.2. Strains and Growth Conditions

The *Candida albicans* strains used in this work are described in Table 1. The strain *C. albicans* ATCC 10231 was used in this study, unless stated otherwise. The *C. albicans* DBT2 (*TOK1*/*tok1*) and DBT3 (*tok1*/*tok1*) strains, obtained by disruption of the *TOK1* gene (37), and the parental strain CAI4 (*TOK1*/*TOK1*) [52] were generously provided by M. Edgerton (University at Buffalo, Buffalo, NY, USA). These strains were grown in YPD (1% yeast extract, 2% peptone, 2% dextrose) medium. *C. albicans* JKC1562 and JKC1594, used for the measurement of cytosolic pH, were kind gifts of J.R. Köhler (Harvard University, Boston, MA, USA) and were grown in SDB medium [53,54]. The strains were aerobically cultured in their appropriate medium for 16–20 h at 30 °C and subcultured to mid-logarithmic growth phase at 30 °C in a rotatory shaker (200 r.p.m.), unless stated otherwise. The cells remained in the yeast (blastospore) phase throughout the study. All yeast strains were stored in YPD or SDB with 20% glycerol at −80 °C and sub-cultured in the respective agar (2%) containing media before each type of test, to ensure optimal growth conditions and purity.

### 4.3. Antifungal Assays

Antifungal activity of lactoferrin was tested in Tris buffer (10 mM Tris-HCl, pH 7.4), unless otherwise noted, as previously described [4]. The water used was from a Millipore Milli-Q Plus 185 ultrapure water system. *C. albicans* cells were cultured in SDB for 16–20 h at 30 °C and subcultured to mid-logarithmic growth phase, washed twice in Tris buffer and resuspended in the same buffer. Cell suspensions (10^5^ cells/mL) were then incubated in the presence or the absence (control) of lactoferrin for 90 min at 37 °C, and aliquots were plated on SDA plates (SDB containing 2% agar). In time-killing kinetic assays, the cells were taken and serially diluted at different intervals after lactoferrin addition and plated on SDA. Unless otherwise specified, cell viability was assessed by colony formation on SDA after 24–48 h incubation at 30 °C. The survival rates were expressed as a percentage of the number of colonies of samples taken before treatments (defined as 100%). The viability of nigericin-treated cells under acidic conditions was determined as described, except yeast cells were grown aerobically in SDB to mid-logarithmic growth phase [40]. Every experiment was performed using two technical replicates and repeated independently at least three times.

### 4.4. Measurement of Extracellular Potassium

Potassium measurements were performed by inductively-coupled plasma optical emission spectrometry (ICP-OES) with a Perkin-Elmer Optima 2000 DV spectrometer using yttrium as the internal standard. Cells were cultured in SDB for 16–20 h at 30 °C, subcultured in SDB to mid-logarithmic growth phase, and rapidly washed in sterile deionized double-distilled water. The cells (10^7^ cells/mL) were immediately incubated with different K^+^ depletion inducers at 37 °C. At indicated time-points, tubes were centrifuged at 1100*× g* for 10 min, and supernatants were collected and stored at 4 °C until analysis. The percentage of cytosolic K^+^ released from cell suspensions treated with 100 μg/mL nystatin was determined in positive control assays. The total K^+^ cellular content (100% value) was measured in the supernatant of cellular suspensions previously treated with 0.5% (*v*/*v*) HClO_4_, heated for 1 h at 95 °C, and centrifuged to remove cell debris. Results are expressed as the percentage (mean ± standard deviation) of K^+^ released relative to the total K^+^ content from duplicates of at least three independent assays.

### 4.5. Glucose-dependent External Acidification

Proton pumping activity of Pma1p H^+^-ATPase was measured by recording the extracellular pH change after the addition of glucose, as previously described [4,55]. Briefly, *C. albicans* cells were grown to mid-log phase in SDB medium, harvested by centrifugation and washed twice in Tris buffer. To determine external acidification, the cells were concentrated (10^7^ cells/mL) in 30 mM or 50 mM KCl solutions and pre-incubated without (control) or with 20 μM lactoferrin for 15 min at 37 °C. The pH of the cell suspensions was adjusted to ~6.7. Proton extrusion was initiated by the addition of glucose (2.5 mM, final concentration) and the time-course was followed using a SevenMulti S50-K pH meter (Mettler-Toledo, Greifensee, Switzerland) with constant stirring.

### 4.6. Measurement of Cytosolic pH

The strain *C. albicans* JKC1562, expressing pHluorin gene from the pJK1027 plasmid, was used to calculate the pH as described, with slight modifications [53,56]. Briefly, the calibration curves were performed using pHluorin-expressing cells, permeabilized with 110 µM monensin and 15 µM nigericin, resuspended in a series of calibration buffers of defined pH (pH 5 to 8) to equilibrate their cytosolic pH. The calibration was carried out by adding 20 µL of cell suspension (OD_600_ = 0.5) to 2 mL of calibration buffer and incubated for 60 min at 30 °C before obtaining measurements. For measuring the cytosolic pH, 100 µL aliquots of cell samples were transferred to a black polystyrene 96-well microtiter plate. The fluorescence of each sample was analyzed with dual excitation wavelengths of 405 nm and 485 nm with a 528 nm emission filter in a Synergy HT Multi-Mode Microplate Reader (BioTek Instruments Inc., Winooski, VT, USA). Fluorescence ratios were recorded, and the cytosolic pH was calculated according to the standard curve. Background fluorescence for a wild-type strain (*C. albicans* JKC1594) not expressing pHluorin was subtracted from the measurements. The data shown are the means from duplicates of at least three independent experiments.

### 4.7. Other Methods

The ionic strength (*I*) of each solution was calculated from the concentrations of ions, according to *I* = ½ Σ *c*_i_ *z*_i_^2^, where *c*_i_ and *z*_i_ are the molar concentration and valence (charge number) of the ionic species (*i*th) in the solution, respectively.

The osmolar concentration of solutions of different osmolarity was determined in triplicate using an automatic osmometer (mod. OM-6060; Arkray Inc., Kyoto, Japan). The osmometer was calibrated against purified water and commercial standard solutions.

### 4.8. Statistical Methods

Data were analyzed by using Prism v.6 software (GraphPad Software Inc., San Diego, CA, USA). Student’s *t* test was carried out on data obtained from at least three independent assays performed in duplicate for each sample. Levels of statistical significance at * *p* < 0.05, ** *p* < 0.01, and *** *p* < 0.001 were used.

## Figures and Tables

**Table 1:** Genotypes and origin of *Candida albicans* strains used in this study.

Strain	Genotype	Ref.
ATCC 10231	Wild type—clinical strain	ATCC
CAI4	*ura3* *::imm434/* *ura3* *::imm434 iro1/iro1* *::imm434* *TOK1/TOK1*	[52]
DBT2	Δ*ura3::imm434/*Δ*ura3::imm434* Δ*tok1/TOK1*	[37]
DBT3	Δ*ura3::imm434/*Δ*ura3::imm434* Δ*tok1/*Δ*tok1*	[37]
JKC1562	*ACT1/_promoter_ACT1-*pHluorin*-NAT1-ACT1his1/his1::tetR-FRT* *arg4/arg4 IRO1/iro1*Δ*::λimm434 URA3/ura3*Δ*::λimm434*	[53]
JKC1594	*ACT1/_promoter_ACT1-NAT1-_terminator_ACT1his1/his1::tetR-FRT* *arg4/arg4 IRO1/iro1*Δ*::λimm434 URA3/ura3*Δ*::λimm434*	[54]
